# Maternal oral health status and preterm low birth weight at Muhimbili National Hospital, Tanzania: a case-control study

**DOI:** 10.1186/1472-6831-7-8

**Published:** 2007-06-26

**Authors:** Elifuraha GS Mumghamba, Karim P Manji

**Affiliations:** 1 Department of Restorative Dentistry, School of Dentistry, Muhimbili University College of Health Sciences, P. O. Box 65014, Dar-es-Salaam, Tanzania; 2 Department of Pediatrics and Child Health, School of Medicine, Muhimbili University College of Health Sciences, P. O. Box 65001, Dar-es-Salaam, Tanzania

## Abstract

**Background:**

The study examined the relationship between oral health status (periodontal disease and carious pulpal exposure (CPE)) and preterm low-birth-weight (PTLBW) infant deliveries among Tanzanian-African mothers at Muhimbili National Hospital (MNH), Tanzania.

**Methods:**

A retrospective case-control study was conducted, involving 373 postpartum mothers aged 14–44 years (PTLBW – 150 cases) and at term normal-birth-weight (TNBW) – 223 controls), using structured questionnaire and full-mouth examination for periodontal and dentition status.

**Results:**

The mean number of sites with gingival bleeding was higher in PTLBW than in TNBW (P = 0.026). No significant differences were observed for sites with plaque, calculus, teeth with decay, missing, filling (DMFT) between PTLBW and TNBW. Controlling for known risk factors in all post-partum (n = 373), and primiparaous (n = 206) mothers, no significant differences were found regarding periodontal disease diagnosis threshold (PDT) (four sites or more that had probing periodontal pocket depth 4+mm and gingival bleeding ≥ 30% sites), and CPE between cases and controls. Significant risk factors for PTLBW among primi- and multiparous mothers together were age ≤ 19 years (adjusted Odds Ratio (^a^OR) = 2.09, 95% Confidence interval (95% CI): 1.18 – 3.67, P = 0.011), hypertension (^a^OR = 2.44, (95% CI): 1.20 – 4.93, P = 0.013) and being un-married (^a^OR = 1.59, (95% CI): 1.00 – 2.53, P = 0.049). For primiparous mothers significant risk factors for PTLBW were age ≤ 19 years (^a^OR = 2.07, 95% CI: 1.13 – 3.81, P = 0.019), and being un-married (^a^OR = 2.58, 95% CI: 1.42 – 4.67, P = 0.002).

**Conclusions:**

These clinical findings show no evidence for periodontal disease or carious pulpal exposure being significant risk factors in PTLBW infant delivery among Tanzanian-Africans mothers at MNH, except for young age, hypertension, and being unmarried. Further research incorporating periodontal pathogens is recommended.

## Background

In Tanzania, the prevalence of severe type of periodontal disease is very low and affects only a minority even though poor oral hygiene is a problem of the majority [1-3]. Severe periodontal disease in the mother has recently been reported to be associated with the occurrence of preterm birth, preterm low-birth-weight (PTLBW) and low-birth-weight (LBW) at full term infant delivery in some populations [4-9]. Furthermore, periodontal pathogens such as *Tannerella forsythia *(previously called *Bacteroides forsythus*), *Porphyromonas gingivalis*, *Actinobacillus actinomycetemcomitans, and Treponema denticola *have been shown to be significantly associated with PTLBW [10-12]. However, contrary findings showing no differences in clinical periodontal status between PTLBW and term normal birth weight outcome have also been reported [11-13].

Potential mechanisms that were put forward to explain the relationship between periodontal disease (PD) and PTLBW infant delivery was that periodontal infection serves as a chronic reservoir of lipopolysaccharide (LPS) which are responsible for the production of interleukin-1beta (IL-1β), prostaglandin E_2 _(PGE_2_), and tumour necrosis factor-alpha (TFNα) that are in turn associated with preterm parturition and fetotoxicity [10,14].

In relation to dental caries in Tanzania, with particular interest to advanced carious lesions presenting as carious pulpal exposure are very common and in most cases the treatment readily available is mainly tooth extraction [15]. Generally, in Tanzania, more women compared to men have more decayed and missing teeth [16]. Also, it has been reported that mothers or pregnant women aged 25 to 49 years in Dar-es-Salaam, have lost an average of two teeth and that more than four teeth were found to have caries [17].

As regards to the microbiology of both dental caries and periodontal disease, some of the microorganisms involved in periodontal lesions such as *Porphyromonas gingivalis, Fusobacterium, Prevotella, Eubacterium, Peptostreptococcus*, and *Spirochetes *were found in endodontic lesions and some of these are responsible for the production of interleukin 1, tumor necrosis factor and prostaglandin [18,19]. Therefore it is considered that, in the event of carious pulpal exposure (CPE), the microbes and their toxins within the pulp tissue (endodontic lesions) would most likely produce interleukin 1, tumour necrosis factor and prostaglandin and exert similar mechanisms responsible for preterm birth as it has been explained for periodontal disease.

Low birth weight (LBW) is a known problem worldwide [20] and appears to be much more pronounced in developing countries. In Tanzania, the prevalence of LBW is about 16%–19% [21,22], whereas among adolescents only, it has recently been reported to be about 48% [23]. From a recent prospective study conducted in antenatal clinics in Mwanza by Watson-Jones and Coworkers, a prevalence of as low as 8% LBW and 12% preterm birth has been reported [24]. In literature, LBW is a multi-factorial problem that incorporates important factors as early pregnancy ≤ 19 years, malaria, anemia, multiple gestations (twins, etc), Edema-proteinuria-hypertension (EPH) ghestosis, infections: urinary tract infections, recurrent bacterial infections, sexually transmitted infections (STI), human immunodeficiency viral (HIV) infection, intestinal parasitic infections, long standing poor nutritional status of the mother, smoking, physically demanding work during pregnancy, and poverty [20,25-28].

Low birth weight infant delivery has been a major health problem associated with high morbidity and mortality rates [22]. Both, periodontal diseases and PTLBW infant delivery are problems that can be prevented [5,18,22]. The association of high prevalence of gingival bleeding, low prevalence of periodontal pockets, as well as a lot of untreated dental caries, particularly carious pulpal exposure with PTLBW among Tanzanians is not known. Bearing in mind the constraints in resources, a retrospective rather than a prospective study was planned. Therefore, the aim of this study was to elucidate the association between oral health status and preterm low birth weight infant deliveries among Tanzanian-African women at Muhimbili National Hospital in Dar-es-Salaam, Tanzania.

## Methods

### Study design, setting and participants

This was a retrospective, unmatched case-control, hospital-based study that involved post-partum (PP) mothers in the maternity block at Muhimbili National Hospital (MNH), Dar-es-Salaam, Tanzania. The MNH is the largest hospital in Tanzania and handles referral patients from all district/municipal hospitals in Dar-es-Salaam region, nearby regional hospitals and other referral hospitals in the country. The PP mothers were those admitted at MNH for various problems of their newborn babies or themselves. The reasons for admission as inquired from mothers included prematurity, birth asphyxia, pneumonia, septicemia, jaundice, malaria, chorioamnionitis, gastro-intenstinal and umbilical cord problems, congenital malformations, anemia in pregnancy, uterus-cervical incompetence, and hypertension. The admissions of the newborns and their mothers were made between day 1 and 31 after delivery (mean 4.2 ± 6.3) with the median and mode being one. The time period that had elapsed between parturition and clinical intra-oral examination of the mother ranged from 1 to 40 days with a mean, median and mode of 8.1 ± 7.4, 5 and 2 days, respectively. The study participants that were examined between 1–7 days after delivery were 62.5%, 8–14 days (22.3%), 15–21 days (7.8%), 22–28 days (4.8%) and 29–40 days (2.7%)), however, the differences between cases and controls in the specified different times of examination were not statistically significant. The PP mothers were categorized into a "case" and "control" as follows: A "case" was defined as a PP mother who had at preterm (gestational age < 37 weeks) live low-birth-weight (PTLBW) singleton delivery weighing < 2,500 gms [20]. Gestational age was based on last menstrual period instead of ultrasound uterine examination, which most mothers cannot afford. A "control" was defined as a PP mother who had at term (gestational age of ≥ 37 weeks) live normal-birth-weight singleton delivery (TNBW) weighing ≥ 2,500 gms. During the six months period of study, a total of 373 consecutive PP mothers (singleton normal deliveries) were recruited for this study (150 cases (PTLBW) and 223 controls (TNBW), a "case:control" ratio of about 1:1.5. Sampling of the participants was not done as all who consented and met the inclusion criteria, were incorporated in the study.

### Inclusion and exclusion criteria

Eligibility to take part in the study was based on the inclusion criteria that qualified any PP mother who delivered a baby or being admitted with a PTLBW or TNBW infant at MNH, provided that the mother had no known underlying systemic disease(s). Screening for HIV was not performed. Exclusion criteria disqualified any PP mother that on medical records had infections or conditions other than periodontal diseases such as genitourinary tract infection, concurrent antibiotic therapy, diabetes mellitus, heart disease, glomerulonephritis, hyperthyroidism, HIV/AIDS, and those with a history of these diseases. This was deemed as necessary because all these conditions were considered to be confounding factors [5,7,10]. Mothers that had multiple pregnancies (twins, etc.) and/or delivered by caesarian-section were also excluded from the study. Eventually, a total of seventy-seven mothers (from both cases and control groups) were excluded from the present report due to the various reasons specified above including those that were "small for gestational age" (SGA), at term low birth weight (TLBW) and preterm normal birth weight (PTNBW) singleton deliveries.

### Data collection: Interview and clinical examination

The interview was conducted by a single person using structured questionnaire to gather information on oral health practices, maternal health status and the related general traditional risk factors for PTLBW infant deliveries, while the interviewer was masked (single-blind) to case-control status. In relation to alcohol consumption, tobacco smoking, exposure to environmental tobacco smoke (ETS) also known as passive or second-hand tobacco smoking the response was set as "yes" or "no" without inquiring for more details regarding quantities, frequency and duration of exposure (e.g. total hours exposed to ETS). A different person (apart from the one conducting interviews) collected specific information on case-control status from the patient's hospital records.

Clinical intraoral examination involved all teeth including third molars, whereby periodontal examination was done on six sites per tooth (buccal-mesial, mid-buccal, buccal-distal, lingual-mesial, mid-lingual and lingual-distal). The tip of the Williams periodontal probe was used to collect plaque from the tooth surface and recorded accordingly as either "present" or "absent". Presence of dental calculus, gingival bleeding on gentle probing, and periodontal probing pocket depth (PPD) was scored separately using the Community Periodontal Index (CPI) clinical probe that has a bulb at the tip (0.5 mm in diameter) with graduations at 3.5 mm, 5.5 mm, 8.5 mm and 11.5 mm [29]. The hierarchical CPI scoring system was not used in this study. A dichotomous scoring system (present or absent) was used for dental calculus and gingival bleeding separately. For periodontal probing pocket depth, categorization of the PPD was done to encompass four different groups (0–3.5 mm, > 3.5 to ≤ 5.5 mm, > 5.5 to ≤ 8.5 mm, and > 8.5 to ≤ 11.5 mm). Gingival recession was scored in millimeters using the "Williams" periodontal probe that had graduations at 1, 2, 3, 5, 7, 8, 9 and 10 mm [30].

Loss of attachment (LA) on tooth surface was computed from the collected clinical data using an indirect approach, "extrapolated loss of attachment (ELA)" as follows: Presence of gingival recession of 1+mm accompanied by PPD 4+mm, operationally was considered as having loss of attachment extending to 5+mm. The operational criteria selected for the clinical definition of study participants who positively and unequivocally exhibited "periodontal disease diagnosis threshold (PDT)" was the presence of both periodontal probing pocket depth of 4+mm in four sites or more and gingival bleeding on gentle probing in 30% or more of the sites that were examined.

Dentition status was assessed according to the methods recommended by the World Health Organization (WHO) [29]. In addition, very advanced carious lesions with carious pulpal exposure were scored separately as follows: Carious pulpal exposure (CPE) – when a tooth had an open deep dentinal carious lesion, with long standing provoked or spontaneous pain that did not subside even after the removal of the irritating factors; signs and symptoms of pulp involvement such as periapical abscess, tenderness to vertical percussion, gumboil, extra-oral facial swelling in close proximity to a carious tooth; carious tooth with polyp; and carious root stumps. Due to constraints in resources, no periapical radiological investigation was performed to confirm the clinical diagnosis.

### Calibration and reproducibility

One calibrated examiner against a "gold standard" examiner performed all the clinical examinations in a single blind to case-control status of the study participants, as the examiner was masked of obstetric data. No calibration was done for gingival bleeding and microbial plaque because for these conditions, it is practically difficult to reach a good reproducible level. During data collection phase, duplicate clinical examinations were done to about 10% (thirty six) of the study participants. Using Kappa test, reproducibility for 6,912 examined sites (kappa value ± standard error, and the 95% confidence interval) for PPD was 0.92 ± 0.01, 0.91 – 0.94), gingival recession (0.78 ± 0.01, 0.76 – 0.81); and 1,152 teeth assessed for scoring dentition status (0.89 ± 0.02, 0.84 – 0.93) and carious pulpal exposure (0.88 ± 0.02, 0.83 – 0.92) whereby in all conditions assessed, the level of significance test was P < 0.001.

### Ethical considerations

The Muhimbili University College of Health Sciences (MUCHS) Research and Publication Committee approved the protocol of this study. The Ethical Committee at MUCHS granted ethical clearance. A written consent was obtained from each study participant before commencement of the interview and clinical examination.

### Data management and statistical analysis

#### Statistical package and level of significance

Data were entered into a personal computer and analyzed using the statistical package for social sciences (SPSS) 10.0 for Windows. Reproducibility calculations are presented as Kappa values with significance levels and the 95% confidence interval (CI). The results are presented as prevalence among cases and controls (for categorical variables) together with Chi-Square test, Odds ratio (OR) and the 95% CI; whereas for continuous variables, the mean values are presented accordingly. For statistical tests, 2-sided type 1 error probability < 0.05 was considered as the level of significance.

#### Transformation of data

During analysis, the number of active tobacco smokers was negligible (three participants only) while there were hundreds of passive or second-hand smokers. These two groups were therefore combined to form one group of "passive smokers" versus those not exposed to tobacco smoke. Also the study participants who qualified for the "ELA" description were categorized as one group against all others with no "ELA" characteristics.

For the purpose of describing the "extent and severity of periodontal disease" in this study population, some principles as described by Carlos and Coworkers for "extent and severity index" were employed [31]. In this study, the "extent of periodontal disease" was expressed in percentages: the number of sites affected with PPD 4 mm or more (4+mm) was the numerator and total number of examined sites was the denominator. The "severity" score for periodontal disease was computed as the mean number of sites with probing periodontal pocket depths 6 mm or more (6+mm) that is the sum of all sites with PPD 6+mm divided by the number of study participants examined.

The "periodontal disease diagnosis threshold (PDT)" categorization was computed as follows: First, the total number of sites in the study participants that exhibited gingival bleeding on gentle probing was counted and those that were ≥ 30% were categorized as one group from those which had < 30% of the sites affected. Secondly, the total number of sites with periodontal pockets 4+mm was counted and the study participants that had four or more sites were categorized as one group from those who had less or equal to three affected sites. Thirdly, the PDT criteria were worked out based on approaches used for PDT elsewhere and the classification of periodontal disease by the American Academy of Periodontology [8,9,32]. The PDT was scored as "yes" if the study participant had exhibited both gingival bleeding in ≥ 30% of the examined sites and probing periodontal pocket depth 4+mm in four or more sites, and "no" for those with lower values than the above set criteria.

In order to find out whether the relationship between periodontal disease threshold and preterm low birth weight is confounded, stratification in the cross-tabulation was done for PDT, PTLBW and other potential confounding factors as recommended by Ylöstalo and Knuuttila [33]. Therefore, a stratum-specific relative risk (RR) estimates resulting from the 2 × 2 stratified cross-tabulations for the association between periodontal disease threshold, preterm low birth weight infant delivery and other potential confounders particularly age, hypertension, number of infant deliveries (parity), history of previous LBW deliveries, heavy duty, cigarette smoking, alcohol consumption and late onset of prenatal care were generated. The potential confounding factors were categorized as either "present" or "absent" (dichotomized). Since the total number for PDT was only 21 study participants, stratification resulted into smaller numbers equal to less than five in a cell of the 2 × 2 table thus necessitating the use of Fisher's Exact test rather than the Chi-Square test.

#### Univariate analysis

The demographic and maternal characteristics included in the univariate analysis were age, education, marital status, tobacco smoking, exposure to environmental tobacco smoke (ETS), alcohol consumption, heavy duty during pregnancy, prenatal care, place of delivery, and parity. Also specific oral health conditions in particular microbial plaque, dental calculus, gingival bleeding (on tooth brushing and as well on gentle probing), periodontal pockets, gingival recession, decayed-missing-filled teeth (independently and as a cumulative index – DMFT) were all incorporated in the univariate analysis and presented accordingly.

#### Multivariate logistic regression model

Multivariate logistic regression model was developed to examine the association between maternal oral health status in particular specific periodontal conditions (periodontal pockets, gingival bleeding, gingival recession, calculus), combined periodontal conditions "periodontal disease threshold", and dentition status (decay-missing-filled teeth and open pulp pulpitis) as risk factors for preterm low birth weight infant delivery. This was done with the understanding that PTLBW is multi-factorial in nature involving demographic, genetic, nutritional, obstetric, antenatal care, oral hygiene, professional dental care, dental plaque, poor oral health, periodontal disease, infection, maternal morbidity and toxic exposure [34] whereby the interaction is not strictly in a hierarchical manner as described by Victoria and Coworkers [35] and presented by Bassani and Coworkers [36]. In the multi-factorial conceptualization, demographic and social factors of importance were age, education, parity, prenatal care, alcohol consumption, tobacco smoking and heavy duty during pregnancy. Environmental tobacco smoke (ETS) was considered as an environmental risk factor for PTLBW. The logistic regression models used were the "Forward stepwise-conditional" based on the likelihood ratio criterion (p_in _= 0.05, p_out _= 0.10) and the "Enter" methods with both "continuous" and "categorical" variables in the model, and in a different phase, a model with "categorical" variables only was developed, accordingly.

##### Model one

The first model analysis included all study participants, both the primi- and multiparous mothers (n = 373), cases (n = 150) and controls (n = 223) whereby the binary dependent variable was PTLBW (yes = PTLBW, no = TNBW) and the covariates were all those that were statistically significant or near the significant level in the univariate analysis. These factors included as covariates were age, marital status, high blood pressure, parity (number of deliveries), frequency of tooth brushing/day, missing teeth, tooth sites that had calculus and gingival bleeding.

##### Model two

In the second model, the analysis included all study participants (n = 373) and variables as in model-one (above), with an addition of new covariates that were considered biologically important based on literature review though not statistically significant in the univariate analysis (forced-entry model). These additional factors were malaria, tobacco smoking and exposure to environmental tobacco smoke (ETS), heavy duty during pregnancy, consumption of alcohol, late antenatal care, home delivery, open pulp pulpitis, periodontal disease threshold, and previous history of low birth weight. Calculus and gingival bleeding were excluded in this model because they are basically in one way or another considered within the periodontal disease threshold.

##### Model three

On the third attempt, the analysis dealt with only the primiparous mothers (n = 206) whereby cases and controls were (n = 92) and (n = 114), respectively. Except for parity that was excluded, the covariates were exactly the same as those included in model-one above.

##### Model four

Again, in this model, analysis dealt with only the primiparous mothers (n = 206), whereby all the covariates in model-two (above) were included, except parity and previous history of LBW, as the study participants were primiparous mothers.

##### Final model

The selected final model was the one that included variables that were statistically significant (P < 0.05) or near significant (P < 0.1) in the univariate analysis. At this stage, in the logistic regression model, the "Enter method" using dichotomized variables based on "yes" or "no" response (e.g. exposure to ETS) and median split values (e.g. for calculus and gingival bleeding) was selected instead of a "combination of both the continuous and dichotomized" variables. There was no "continuous variable model" for the whole data set, due to the fact that the type of data collected from the study population did not improvise for such transformation.

## Results

The following are the results based on 373 inpatient postpartum mothers. None of the participants had been exposed to oral health education or periodontal therapy before the study.

### Demographic background characteristics

The demographic background characteristics of the study participants are shown in Table 1. Mothers that were un-married were about two times more likely to deliver a PTLBW than those who were married (OR = 1.9, 95% CI = 1.2, 2.9; P = 0.004). Among the 106 teenage-mothers (14–19 yrs), there were more un-married mothers (59, (55.7%) than married ones (47, (44.3%), (P = 0.042). Teenage-mothers that were un-married were almost three times more likely to have PTLBW as compared to the married ones (OR = 2.9, 95% CI = 1.4, 5.8; P = 0.003). Infant delivery at teenage (≤ 19 years of age) were about two and a half more likely to be a PTLBW when compared to deliveries from mothers aged ≥ 20 years (OR = 2.4, 95% CI = 1.5, 3.8; P < 0.001).

**Table 1:** Demographic characteristics of the study participants in percentages and Odds ratio

Demographic characteristics	Cases (PTLBW) n (%) 150 (40.2)	Controls (TNBW) n (%) 223 (59.8)	Crude Odds ratio (95% CI)	P-value
Marital status				
Not married	67 (44.7)	67 (30.0)	1.88	0.004
Married	83 (55.3)	156 (70.0)	(1.22–2.89)	
Tobacco smokers				
Yes	2 (1.3)	1 (0.4)	3.0	0.567*
No	148 (98.7)	222 (99.6)	(0.27–33.38)	
Passive/2^nd ^hand smokers				
Yes	91 (60.7)	137 (61.4)	0.97	0.881
No	59 (39.8)	86 (38.6)	(0.63–1.48)	
Alcohol consumption				
Yes	23 (15.3)	33 (14.8)	1.04	0.887
No	127 (84.7)	190 (85.2)	(0.59–1.86)	
Late onset of prenatal care				
Yes	121 (80.7)	183 (82.1)	0.91	0.733
No	29 (19.3)	40 (17.9)	(0.54–1.55)	
Delivered at home				
Yes	5 (3.3)	3 (1.3)	2.53	0.276*
No	145 (96.7)	220 (98.7)	(0.60–10.74)	
Delivery at teenage (≤ 19 yrs)				
Yes	59 (39.3)	47 (21.1)	2.43	< 0.001
No	91 (60.7)	176 (78.9)	(1.53–3.84)	
Heavy duty during pregnancy				
Yes	10 (6.7)	11 (4.9)	1.38	0.476
No	140 (93.3)	212 (95.1)	(0.57–3.33)	
Education: ≤ Primary				
Yes	131 (87.3)	188 (84.3)	1.28	0.415
No	19 (12.7)	35 (15.7)	(0.70–2.34)	
Use of coffee				
Yes	18 (12.0)	38 (17.0)	0.66	0.181
No	132 (88.0)	185 (83.0)	(0.36–1.21)	

*Fisher's Exact Test

The study participants that were active tobacco smokers were very few (only 3 mothers out of 373 participants (0.8%)), but were accompanied by a substantial proportion of those exposed to ETS at home or at work place (61.1%). Alcohol consumption during pregnancy was remarkable (15%) and late onset of prenatal care (≥ 24^th ^week of pregnancy) was very high both in controls (82%) and in cases (81%). However, the differences between cases and controls on these known risk factors for PTLBW: active smoking (OR, 3.0), alcohol use (OR, 1.04) and late onset of prenatal care (OR, 0.9) did not reach a statistically significant level. When comparing late to early onset of prenatal care, late onsets had higher proportion of individuals: with low level of education, primary or no education at all (87.5% versus 76.8%, P = 0.023), who were un-married (38.5% versus 24.6%, P = 0.030), and who were primiparous (47.7% versus 31.9%, P = 0.017). There were eight (2.1%) unplanned home deliveries, which were afterwards reported and hospitalized. Although not statistically significant, mothers who delivered at home were two and a half times more likely to have PTLBW than those who delivered in hospital (OR = 2.5, 95% CI = 0.6, 10.7; P = 0.276).

### Distribution of clinical characteristics

The distribution of specific clinical characteristics among cases and controls is shown in Table 2. The differences in the distribution of periodontal pockets 4+mm among cases (28.7%) and controls (30.0%), (OR = 0.9, 95% CI = 0.6, 1.5) and also periodontal pockets of 6+mm in cases (3.3%) and in controls (2.7%), (OR = 1.2, 95% CI = 0.4, 4.2) were not statistically significant. Using "ELA" approach, there were 192 (51.5%) participants that had both gingival recession 1+mm and periodontal pockets 4+mm. The proportion of study participants with "ELA" among cases and controls were 52.0% and 51.1% respectively (OR = 1.04, (95% CI: 0.69, 1.57) but the difference was not statistically significant.

**Table 2:** Clinical characteristics of the study participants in percentages and Odds ratio

Clinical characteristics	Cases (PTLBW) n (%) 150 (40.2)	Controls (TNBW) n (%) 223 (59.8)	Crude Odds ratio (95% CI)	P-value
Periodontal pockets (PK) 4+mm				
Yes	43 (28.7)	67 (30.0)	0.94	0.775
No	107 (71.3)	156 (70.0)	(0.59–1.48)	
Periodontal pockets (PK) 6+mm				
Yes	5 (3.3)	6 (2.7)	1.25	0.719
No	145 (96.7)	217 (97.3)	(0.37–4.16)	
Gingival recession				
Yes	64 (42.7)	81 (36.3)	1.31	0.218
No	86 (57.3)	142 (63.7)	(0.85–1.99)	
Gum bleeding on brushing				
Yes	56 (37.3)	81 (36.3)	1.04	0.843
No	94 (62.7)	142 (63.7)	(0.68–1.60)	
Extrapolated Loss of attachment (ELA)				
Yes	78 (52.0)	114 (51.1)	1.04	
No	72 (48.0)	109 (48.9)	(0.69–1.57)	0.868
Periodontal disease diagnosis Threshold: GB ≥ 30% sites, PK 4+ mm in ≥ 4 sites (PDT)				
Yes	11 (7.3)	10 (4.5)	1.69	0.242
No	139 (92.7)	213 (95.5)	(0.70–4.08)	
Decayed teeth				
Yes	76 (50.7)	119 (53.3)	0.90	0.609
No	74 (49.3)	104 (46.6)	(0.59–1.36)	
Carious pulpal exposure				
Yes	28 (18.7)	44 (19.7)	0.93	0.798
No	122 (81.3)	179 (80.3)	(0.55–1.58)	
Decayed-Missing-Filled Teeth				
Yes	91 (60.7)	142 (63.7)	0.88	0.556
No	59 (39.3)	81 (37.5)	(0.57–1.35)	
High BP during pregnancy				
Yes	22 (14.7)	16 (7.2)	2.22	0.019
No	128 (85.3)	207 (92.8)	(1.13–4.39)	
Malaria during Pregnancy				
Yes	91 (60.7)	126 (56.5)	1.19	0.424
No	59 (39.3)	97 (43.5)	(0.78–1.81)	
Primiparous (1^st ^child delivery)				
Yes	58 (38.7)	109 (48.9)	0.66	0.052
No	92 (61.3)	114 (51.1)	(0.43–1.00)	
Previous LBW				
Yes	8 (5.3)	8 (3.6)	1.5	0.415
No	142 (94.7)	215 (96.4)	(0.56–4.13)	
Previous abortion				
Yes	31 (20.7)	44 (19.7)	1.06	0.825
No	119 (79.3)	179 (80.3)	(0.63–1.77)	

According to the operational criteria for periodontal disease diagnosis threshold, the proportion of study participants that had gingival bleeding in ≥ 30% of the examined sites were 79.3% and 71.3% in cases and controls respectively (OR = 1.5, 95% CI = 0.95, 2.52, P = 0.081); and those that had periodontal pockets 4+mm in ≥ 4 sites were 24 only (6.4%), among cases (13/150, (8.7%) and in controls (11/223, (4.9%), (OR = 1.8, 95% CI = 0.80, 4.20, P = 0.150). Therefore, study participants who positively and unequivocally exhibited periodontal disease diagnosis threshold, i.e. those who had both gingival bleeding ≥ 30% of the examined sites and periodontal pockets in ≥ 4 sites) were 21 only (5.6%). The proportion of study participants who fulfilled the PDT criteria was slightly higher among cases (7.3%) than in controls (4.5%), but the difference did not reach a level that was statistically significant (OR 1.7 (95% CI: 0.70, 4.08), P = 0.242).

The distribution of clinically determined carious pulpal exposure (with no confirmation by radiological investigation) was 18.7% among cases and 19.7% in controls (OR = 0.9, 95% CI = 0.6, 1.6; P = 0.985). Among the clinical features that were statistically significant was high blood pressure (≥ 140/90 mmHg) during pregnancy and participants that were in this category were almost two times more likely to have PTLBW than those with normal blood pressure: OR = 2.2, 95% CI = 1.1, 4.4; P = 0.019). Other factors such as being primiparous, previous history of LBW, and clinical malaria during pregnancy were not statistically significant. Regarding tooth extraction during pregnancy, four study participants (1.1%); one among cases and three in control group (P = 0.651) had it done before parturition (data not in Table 2).

The mean age of the PTLBW mothers was significantly lower than the TNBW mothers (22.5 ± 5.6 versus 23.9 ± 5.2, P = 0.013, respectively). After having examined all teeth in the oral cavity, the proportion of study participants that had third molars was found to be 83.4%. The mean number of sites that exhibited gingival bleeding on gentle probing was significantly higher in cases (80.6 ± 35.4) than controls (72.5 ± 34.3, P = 0.026). Although not statistically significant, PPD 4+mm sites were slightly higher among cases (0.93 ± 2.96) than controls (0.78 ± 2.15), (P = 0.552). The mean number of sites that had gingival recession 1+mm was almost the same in cases (131.83 ± 28.43) and in controls (131.57 ± 28.80), (P = 0.242). Having done the stratification of the potential confounders and in particular the age, hypertension, parity, previous LBW delivery, heavy duty during pregnancy as well as cigarette smoking, alcohol consumption and late onset of prenatal care, none of the factors did reach a level that was statistically significant (data not shown).

### Multivariate logistic regression model – adjusting for the known risk factors

Detailed analysis while adjusting for specific factors in the logistic regression model using primi- and multiparous postpartum mothers together (n = 373) is shown in Table 3. Factors that were statistically significant or near significant level in the univariate analysis were included in the model and these were age, marital status, hypertension during pregnancy, parity, tooth brushing practice (frequency), calculus, gingival bleeding, and missing teeth (model one). Among these, factors that were likely to pose an increased risk for PTLBW were maternal age ≤ 19 years (OR^a ^= 2.09, 95% CI = 1.18–3.67, P = 0.011), hypertension during pregnancy (OR^a ^= 2.44, 95% CI = 1.20–4.93, P = 0.013), and being un-married (OR^a ^= 1.59, 95% CI = 1.00–2.53, P = 0.049). When a forced entry method was used (Table not shown) whereby additional covariates that were thought to be biologically important were incorporated in the model (model two), significant risk factors for PTLBW were maternal age ≤ 19 years (OR^a ^= 2.54, 95% CI = 1.18–3.67, P = 0.001), hypertension during pregnancy (OR^a ^= 2.44, 95% CI = 1.19 – 5.02, P = 0.015), and being un-married (OR^a ^= 1.69, 95% CI = 1.05 – 2.71, P = 0.049). In the latter model (model two), though it did not reach a significant level, home deliveries were more likely to be PTLBW than hospital ones (OR^a ^= 4.61, 95% CI = 1.00 – 21.22, P = 0.050).

**Table 3:** The multivariate logistic regression model of all preterm low-birth-weight mothers

Risk factors for PTLBW	All preterm low birth weight infant deliveries (PTLBW), using primi- and multiparous mothers together (n = 373)		
	
	Adjusted Odds ratio	95% CI	P-value
Age (≤ 19 years)	2.09	1.18 – 3.67	0.011
Marital status (not married)	1.59	1.00 – 2.53	0.049
Hypertension during pregnancy	2.44	1.20 – 4.93	0.013
Primiparous (1^st ^child delivery)	0.96	0.57 – 1.61	0.871
Tooth brushing practice – less frequent	1.32	0.84 – 2.06	0.224
Calculus	1.37	0.83 – 2.26	0.219
Gingival bleeding	1.06	0.64 – 1.74	0.834
Missing teeth	1.12	0.72 – 1.76	0.615

The findings for the logistic regression model (model three) using primiparous mothers only (n = 206) are shown in Table 4. Significant risk factors for PTLBW among primiparous mothers were maternal age ≤ 19 years (OR^a ^= 2.07, 95% CI = 1.13 – 3.81, P = 0.019), and being un-married (OR^a ^= 2.58, 95% CI = 1.42 – 4.67, P = 0.002). When biologically important variables (Table not shown) were slotted-in in the forced entry model for primiparous study participants (model four), the most important risk factors for PTLBW persisted to be maternal age ≤ 19 years (OR^a ^= 2.72, 95% CI = 1.47 – 5.05, P = 0.002), and being un-married (OR^a ^= 2.43, 95% CI = 1.32 – 4.47, P = 0.004).

**Table 4:** The multivariate logistic regression model of primiparous preterm low-birth-weight mothers

Risk factors for PTLBW	Primiparous preterm low birth weight (PPTLBW), infant deliveries, using all primiparous mothers only (n = 206)		
	
	Adjusted Odds ratio	95% CI	P-value
Age (≤ 19 years)	2.07	1.13 – 3.81	0.019
Marital status (not married)	2.58	1.42 – 4.67	0.002
Hypertension during pregnancy	2.17	0.76 – 6.23	0.151
Tooth brushing practice- less frequent	1.28	0.69 – 2.36	0.430
Calculus	1.21	0.61 – 2.39	0.585
Gingival bleeding	1.29	0.65 – 2.54	0.47
Missing teeth	1.43	0.75 – 2.74	0.281

### The "extent and severity" of periodontal disease

A total of 63,759 periodontal sites were clinically examined for periodontal disease. Regarding "extent" of the problem of periodontal disease is that, PPD 4+mm were found in 140 out of 25,658 (0.55%) sites among cases, and in 173 out of 38,101 (0.45%) sites among controls (OR = 1.2, 95% CI: 0.96, 1.15) but the differences between cases and controls were not statistically significant (P = 0.105). The "severity" score for periodontal disease was 0.13 in cases and 0.11 among controls, P = 0.451).

## Discussion

A number of features inherent in this case-control study deserve some clarification. For convenience, the study was conducted in the maternity block at the MNH, the place where mothers with PTLBW infants can readily be found. Therefore, cases and controls were recruited from the same place. Due to constraints in resources and the set-up of prenatal clinics, it was difficult to recruit pregnant mothers for interviews and clinical examinations at labor wards before delivery, particularly when considering comfort. For this reason it was regarded as much more appropriate to recruit inpatient post-partum mothers. The study participants were interviewed on the reasons for their admission to the hospital but the information was not used to categorize them into cases and controls. Majority of them had low level of education and did not know exactly the diagnosis of their problems and it is more often taken as a tradition to keep those fine details with the medical practitioners. Therefore, patient's medical records were used to get information on weight of the infants and maternal gestation age at delivery. The criterion used to categorize low birth weight and preterm birth is the one that has been in wide use as recommended by WHO and has previously been applied in Tanzanian setting, therefore, data can be compared broadly [20,37].

The occurrence of teenage pregnancies was substantial and unplanned home deliveries were few both of which are consistent with earlier findings [38]. In the univariate as well as logistic regression analysis, un-married mothers were more likely to deliver PTLBW compared to those who were married and the risk was more pronounced to about one and a half times in the primiparous mothers. The reason for this finding is not yet established but it is speculated that unmarried mothers might have stress and experience long standing poor nutritional status due to wide spread poverty, factors known to contribute to low birth weight [20,27,34,39]. The effect of hypertension, which appeared to be a significant risk factor for PTLBW among all studied mothers (primi- and multiparous together), was not significant when primiparous mothers were considered separately. It is thought that age might be a contributing factor. When mothers that had PTLBW were compared to TNBW mothers, the PTLBW had a lower mean age, which is in agreement with other findings from elsewhere for example in USA [6]. In this study teenage mothers were more at risk for PTLBW than mothers that were 20 years of age or more and this also is in agreement with what has been found elsewhere [34].

The prevalence of periodontal conditions in this study population represents the natural history of periodontal disease because the participants had no history of being exposed to either oral health education or periodontal therapy. Despite, the relatively high occurrence of microbial plaque, dental calculus and gingival bleeding on probing, the prevalence of severe periodontal disease was low and corroborates with other findings in Tanzania, and neighboring country, Kenya [1-3,40,41]. Moreover, majority of the populations in Tanzania and Kenya retained most of their dentition in a functional state even up to the age of 65 years signifying that severe destruction of periodontal tissue is minimal [42,43].

The time period that had elapsed between parturition and periodontal examination was close to the previously reported time of hospital stay (one to thirty eight days and a mean of about eleven days) for the LBW infants [22]. However, this elapsed time possibly might have resulted to an underestimation of the gingival bleeding scores due to gradual resolution of gingival inflammation, a phenomenon that is common within two to three months after delivery [44,45]. The finding that PTLBW had significantly higher mean number of sites with gingival bleeding on probing as compared to the TNBW is in agreement to what was found among Caucasian mothers in Brazil [8].

Although periodontal disease profile differ from one population to another, it had earlier been reported that the differences do not conform to the traditional generalization that African and Asian populations suffered more severe periodontal disease than other populations [36,46]. The occurrence of severe periodontal disease (6+mm) among mothers at MNH was low (about three percent) compared to ten percent among Saudi mothers whereby periodontal disease accounted for four times increased risk for PTLBW [7]. This suggests that there might be some racial and regional differences in the prevalence and risk factors for PTLBW infant deliveries. The indirect approach used to generate information on loss of attachment, "ELA", in this study did not show any significant difference between preterm and term deliveries, and the findings are contrary to what has been reported in the study on "oral conditions and pregnancy (OCAP)" in USA that used a direct approach to assess loss of attachment [6].

In this study, the proportion of participants exposed to ETS was substantial while those exposed to active or direct tobacco smoke was negligible. Information on smoking and alcohol consumption during pregnancy among Tanzanian women is scarce. Exposure to ETS in this study did not appear to be a significant risk factor for PTLBW and is in line with Steyn and Coworker's findings [47]. However, it disagrees with the findings of Windham and Coworkers whereby the exposure time to ETS was set at ≥ 7 hours/day in non-smokers [48]. Since in our study there were extremely few active tobacco smokers and the difference between cases and controls was not significant, this does not contradict in any way other reports that active heavy tobacco smokers (ten or more cigarettes/day) are at higher risk for PTLBW [20]. The intensity of tobacco smoking during pregnancy among Tanzanian-African women has been stated to be two cigarettes per day (unpublished).

It was interesting to note that despite the good and accessible health facilities starting from dispensaries, health centres, district hospitals as well as regional and referral hospitals in urban areas and in particular Dar-es-Salaam, most of the participants, both "cases" and "controls", started prenatal clinic late. The reason for this behavior is not known, though it was observed that late attendees of prenatal care had higher proportion of individuals who were primiparous, un-married and have low level of education. Ignorance, lack of experience and fear among those who were not married might have contributed to the late reporting for prenatal care. Such high rate of late reporting for prenatal care has also been reported among other maternal populations in Mwanza, Tanzania [24].

The findings that both smoking as well as late prenatal care did not appear to be significant risk factors for PTLBW in this study population are in agreement with other reports by Moliterno and coworkers in Brazil [9] as well as Buduneli and collaborators in Turkey [49]. In Brazil and Turkey studies the prevalence of destructive chronic periodontal disease was low and therefore comparable to the one reported in our present study. However, lack of significance for late prenatal care versus occurrence of PTLBW is contrary to what has been reported in relation to either LBW or PTLBW [5,20,36] and the possible reason might be attributed to the socioeconomic and nutritional status [23] of the study participants and quality of prenatal care, factors that were not within the scope of the present study. Information based on pregnant mothers attending prenatal care in three reproductive clinics in Dar-es-Salaam showed that active smoking in pregnancy was negligible (0.3%) but ETS was substantial (43%) and that alcohol consumption was 35% (unpublished). None of the pregnant women did take alcohol on daily basis. Mostly they used alcohol on weekends and the amount was 1–2 drinks per week. It is speculated that the study participants at MNH had similar pattern of alcohol consumption, because most of them if not all were also from Dar-es-Salaam. Low or moderate consumption of alcohol on weekends in some populations elsewhere has not been associated with an increased risk for low birth weight [50]. The amount of alcohol consumption that has unfavorable outcome on pregnancy, either preterm birth or small-for-gestation-age (SGA) has been reported to be 3 or more drinks per day [51,52].

From previously studied population in Tanzania, it has been reported that in about two thirds of the mothers that had low birth weight infants, no associated maternal factors could be detected [28]. After stratification of the potential confounding factors [33], some factors had higher relative risk where confounders were "present" compared to where they were "absent", but none of them appeared to be significant risk factors for PTLBW. This does not mean at all that there were no clinical significance in the factors studied, but rather it underscores need for a more detailed study that will include among others, wider scope of PTLBW potential risk factors, intensity and duration of the exposure.

In relation to ethnicity, the present study population exclusively involved indigenous Tanzanian-African women. In some reports where periodontal disease has been shown to be one of the significant risk factors for PTLBW, majority of the participants were Blacks/African-Americans [4,6]. However, based on clinical findings from the present study population, there was no evidence to support the notion that maternal periodontal disease is a significant risk factor for PTLBW. This corroborates with other findings elsewhere [53,54], including women of low socioeconomic status [49], and those who never smoked tobacco [55]. The possible explanation for this might be that different populations may be subject to different risk factors for the development of a specific pathology. Furthermore, some important periodontal pathogens have significantly been associated with PTLBW [11,12,56]. Since this was beyond the scope of the present study, it remains a subject for further research in Tanzania, preferably using most modern techniques such as checkerboard DNA-DNA hybridization and polymerase chain-reaction (PCR) techniques [10,11,19,49].

The level of dental caries, expressed as independent components as "tooth-decay (D), missing (M), filling (F), and in a cumulative index as DMFT, and carious pulpal exposed lesions, all appeared to be not significant risk factors for the occurrence of PTLBW. The findings are in agreement with what has been reported from Thailand [5] and Finland [54]. However, lack of evidence in the present study that poor periodontal or dentition status may be a significant risk factor for PTLBW, does not preclude in any way the importance of pregnant mothers to have routine dental check-ups and management as recommended by other authorities because this will arrest the progression of the disease [13,53,57]. However, recently also, it has been reported in large randomized trials that treatment of periodontal disease in pregnant women did not change the outcome rates of preterm birth or low birth weight [58]. Even in occasion where the treatment was interrupted, it did not lead to an increased risk for LBW infant when compared to women with no history of periodontal care [59].

Among the limitations of the present study worthy of commenting are: One; the human immunodeficiency Virus (HIV) and helminthic infections that have recently been reported to be associated with higher risk of low birth weight [25,27], and nutritional status of the mother were not assessed because they were not within the scope of the present study and therefore their impact on this study is not known. Two; the diagnosis of carious pulpal exposure was based on clinical presentation alone and was not confirmed with radiological examination nor was there any microbiological investigation for the presence of periodontopathic bacteria in the involved teeth and hence this might have threatened the validity of this measurement. Three; the study was done at the maternity block in a government national hospital situated in Dar-es-Salaam city and therefore the results cannot be generalized to include for example rural population that has been reported to have a slightly worse periodontal health compared to urban residents [3].

## Conclusions

It is concluded that, on the basis of these clinical findings on periodontal conditions and dentition status, there is no evidence to support the notion that periodontal disease or carious pulpal exposure are significant risk factors for preterm low-birth-weight infant delivery among Tanzanian-African mothers at MNH. Significant risk factors for PTLBW were young age, hypertension, and being un-married. Further research that is prospective in nature and that will incorporate periodontal pathogens and mediators of inflammation in a larger representative sample of pregnant women is needed.

## Competing interests

The author(s) declare that they have no competing interests.

## Authors' contributions

EGM: Principal investigator conceived and designed the study, supervised data collection, managed data, statistical analysis, organization and preparation of the manuscript.

KPM: Co-investigator participated in designing the study, field organization and preparation of the manuscript. Finally, all authors, read, agreed to its contents and approved the final manuscript in its present form.

## Pre-publication history

The pre-publication history for this paper can be accessed here:


